# Expected reasons for leaving the labour market and loss of paid employment among older workers: prospective cohort study

**DOI:** 10.1186/s12889-023-15242-5

**Published:** 2023-03-17

**Authors:** Emil Sundstrup, Annette Meng, Sebastian Venge Skovlund, Karen Albertsen, Lars L. Andersen

**Affiliations:** 1 National Research Centre for the Working Environment, Lersø Parkallé 105, 2100 Copenhagen, Denmark; 2 Department of Sports Science and Clinical Biomechanics, University of Southern Denmark, Odense, Denmark; 3 TeamWorkingLife, Hoeffdingvej 22, 1, Valby, 2500 Denmark

**Keywords:** Senior workers, Ageing, Public health, Retirement, Sustainable employment

## Abstract

**Background:**

Surveying expected reasons for retirement may be a useful strategy to maintain labor market affiliation. The aim was to investigate the prospective association between self-reported expected reasons for leaving the labour market and subsequent loss of paid employment before the state pension age among older workers.

**Methods:**

The prospective risk of loss of paid employment before the official state pension age was estimated from expected reasons for leaving the labour market among 10,320 currently employed older workers (50–63 years) from the SeniorWorkingLife study. In 2018, participants replied to 15 randomly ordered questions about expected reasons for leaving the labour market and were in 2020 followed in a national register containing information on labour market participation.

**Results:**

Loss of paid employment before state pension age was predicted by expected reasons related to ‘Health, work demands and occupational well-being’: ‘Poor physical health’ (RR 1.47, 95% CI 1.45–1.49), ‘Poor mental health’ (RR 1.36, 95% CI 1.32–1.40), ‘Not being capable of doing the job’ (RR 1.20, 95% CI 1.18–1.22), and ‘Not thriving at the workplace’ (RR 1.14, 95% CI 1.11–1.17). Expected reasons related to the possibility of receiving voluntary early retirement benefits also increased this risk. Expected reasons related to ‘Leisure’ (‘Wish for more self-determination’; ‘Wish for more time for hobbies’), ‘Economy’ (‘Economic considerations’; ‘Possibility of receiving pension’), and ‘Norms’ (‘Retirement norms’; ‘To make space for younger employees’) decreased the risk of loss of paid employment before state pension age. Age-stratified analyses revealed that expected reasons related to the domain of ‘Health, work demands and occupational well-being’ predicted risk of loss of paid employment to a greater extent among workers aged 50–55 compared to those aged 56–63.

**Conclusions:**

Expected reasons for leaving the labour market predicted actual labour market participation among older workers in Denmark. Expected reasons related to poor physical and mental health, and not being capable of doing the job seem to be stronger PUSH-factors among workers aged 50–55 compared to those aged 56–63. Preventing early labour market detachment should take the worker’s expected reasons for leaving into account.

## Background

As a consequence of demographic changes, in particular a growing proportion of older people, European countries are introducing pension reforms to increase the labour market participation of older workers. In Denmark, the state pension age is gradually increasing and retiring early has become increasingly difficult. However, poor health and unfavourable working conditions may constrain keeping older workers longer in the labour market, and for some older workers, coping with the demands of an extended working life may be challenging. Early identification of individuals at risk for premature exit from the labour market could be crucial for designing and targeting preventive workplace efforts and societal campaigns.

Expected reasons for retirement and the actual transition to retirement is a dynamic and complex process influenced by factors related to both the individual, the workplace, and the labour market and its policies [1, 2]. Factors stimulating older workers to leave the labour market before the official state pension age are known as push, pull, and jump mechanisms [3, 4]. Push refers to involuntary labour market exit where people are being ‘pushed’ out, for example, due to poor health. Pull is defined as voluntary early retirement instigated by attractive early retirement schemes or retirement norms, i.e. when it is appropriate to leave the labour market. Jump refers to voluntary early retirement initiated by personal needs and values, e.g. spending time with grandchildren or the desire to travel the world [3, 5].

Poor health is an established push factor and has previously been associated with risk of sickness absence and early retirement [1, 6–9]. In line with this, a meta-analysis found poor physical and mental health as predictors for early retirement, underscoring poor health as an important determinant of premature exit from the labour market [1]. In addition, several work environmental factors have been identified as either push or stay factors. High physical work demands are push factors that have been found to associate with increased risk of sickness absence [10–13], early retirement [9, 14, 15], and disability pension [15–17] especially among older workers [18]**.** Adverse psychosocial working conditions can also act as push factors, evidenced by studies where low job control [16, 19–21] and low skill discretion [14] predicted early retirement. By contrast, positive psychosocial work factors such as high influence and recognition from management act as stay factors that increase the likelihood of choosing to work beyond state pension age [22]. Research has also pointed towards a relationship between personal economy and the intention to retire or continue working. For instance, a recent study among senior workers identified financial incentives (i.e. if it would pay off better economically) as one of the most prevalent expected reasons to prolong working life [23]. Access to early retirement benefits through a voluntary early retirement program has also been shown to predict early retirement among older Danish workers [24]. Early retirement benefits have, therefore, been considered a factor that pulls people out of the labour market, but may also be considered a way out of the labour market for those experiencing strong push factors, e.g. poor health or exposure to adverse physical and psychosocial working conditions [24–26]. Existing studies of retirement patterns of older workers have shown that intentions to retire are conditioned by a preference for more leisure time to be used for a new ‘life project’ and/or social gains such as spending more time with grandchildren [27]. In line with this, wish for more time for leisure activities has previously been recognized as a jump factor and as a source of motivation for retirement [28]

Asking older workers, years before state pension age, about expected reasons for leaving the labour market, could potentially provide useful knowledge for implementing effective workplace strategies aiming at increasing or maintaining future labour market participation. In a cross-sectional design, we have previously investigated factors conditioning retirement intentions by analyzing replies from more than 11,000 older workers (≥ 50 years) to questions on expected reasons for leaving the labour market [3]. That analysis showed that poor physical health and not being capable of doing the job are common reasons for expecting to retire early among older workers with physically demanding work. The predictive value of these questions should, however, be assessed in a prospective study design, before workplaces can reliably and validly use and act on this information. Thus, our knowledge about factors conditioning intentions to retire—and how these factors relate to future labour market participation—is far from complete. Such knowledge could help identify vulnerable groups of older workers and initiate preventive efforts in the workplace aiming at increasing or maintaining future labour market participation.

Previous studies on retirement intentions have mainly focused on factors conditioning these intentions and not whether retirement intentions in fact predict actual behaviour later in life (i.e. labour market participation). And studies have mainly been on particular occupational groups, e.g. physicians, practitioners, professional workers or older workers on an aggregate level and not a sample representative of the general working population of older workers. In addition, older workers are defined quite broadly with regard to the age span, and it could be speculated, that the differences between 50-year-old and 60-year-old workers with regard to expected reason for retirement – and its association with future labour market participation – could be quite diverse.

Therefore, we aim to address these research gaps by investigating the prospective association between expected reasons for retirement and subsequent loss of paid employment before the state pension age among older workers. To investigate this, the current analysis combines representative survey data from Danish employed individuals (50–63 years) with a national register containing information on labour market participation. To evaluate potential age differences, this study recruited a large group of older employees (+ 10.000), which allowed for stratification into two distinct age groups: 50–55 years and 56–63 years.

## Methods

### Study design

This prospective cohort study merges survey data on expected reasons for leaving the labour market from the first wave of the SeniorWorkingLife study (identification number in ClinicalTrials.gov: NCT03634410) with register information on labour market participation [29]. A total of 18,000 employed Danes ≥ 50 years were invited to participate in the SeniorWorkingLife study and received a personal survey link in e-Boks (an online digital mailbox linked to the Danish social security number). In case of non-response, two digital reminders were sent within the collection period and participants also received a reminder by telephone. Baseline data collection was terminated in October 2018 and among the invited employees, 56% answered all the survey questions (i.e. those who also answered other questions about the working environment and health, which are not included in this article). The participants who only partially answered the survey – but responded fully to the questions regarding expected reasons for leaving the labour market (described below)—were also included in the analyses. Furthermore, we only included participants who confirmed in the baseline survey that they were currently employed wage-earners and the study population was truncated at the age of 63 at baseline to ensure that the included participants could not have left the labour market at or after the official state pension age of 65 years during the follow-up period. This yield a total study sample of 10,320 employed older workers replying in 2018 and with follow-up information from registers in 2020. Since outcome data was register-based and not survey-based, no participants were lost to follow-up.

Currently employed wage earners were defined based on previously described criteria [29]: 1) the person should have been in paid employment for at least 20 h per week during March 2018 and for at least half of the months during the last year as of March 2018, and 2) the person was not allowed to have received economic benefits from ‘flexible jobs’ (a job offer on special terms for people with permanently reduced work ability), “light jobs” (work on special terms with a wage subsidy offered to people on a disability pension), sickness absence, or maternity/paternity leave during the first quarter of 2018.

More information on the SeniorWorkingLife study can be found in the previously published protocol paper [29].

### Predictors

First, participants were asked about their expected retirement age. Next, participants replied to a multiple-choice questionnaire battery on factors potentially conditioning retirement intentions. These factors are in full length described elsewhere [3, 23]. But, briefly summarized, the questionnaire contained 15 multiple-choice response options— provided in random order—concerning expected reasons that might cause the respondents to leave the labour market:

(i) Push: poor physical and mental health; that you do not thrive at the workplace; termination of employment relation; at the request of the workplace; that you will not be capable of doing your job; to make space for younger employees.
(ii) Pull: retirement norms (it is common to leave at that age in your type of work); economic considerations, including the possibility of receiving voluntary early retirement or pension benefits; good retirement conditions at the workplace.
(iii) Jump: wish from spouse; wish for more self-determination (that you want to determine yourself what you want to do); wish for more time for hobbies/leisure activities.

The option ‘None of the above’ was given at the bottom of the multiple-choice questions as the 16th option. An overview of the response options, ordered by domain (i.e. ‘Leisure’, ‘Health, work demands and occupational well-being’, ‘Economy and retirement considerations’, ‘Norms’, ‘External factors’[3]), can be seen in Table 2.

### Outcome

The outcome variable ‘loss of paid employment’ from 2018 to 2020 was assessed by Statistics Denmark based on national registers. Loss of paid employment was defined as those who, in March 2020, no longer fulfilled the criteria of being employed (similar criteria as described in ‘Study Design’, but two years later) [30]. Importantly, the study population was truncated at the age of 63 at baseline, meaning that the included participants could not have left the labour market at or after the official state pension age during the follow-up period [30]. In 2018, the state pension age was 65 years, but this was raised to 66 years in 2020. Thus, participants aged 63 years could not reach more than 65 years at follow-up, i.e., before state pension. Thus, the outcome variable defines loss of paid employment before the state pension age.

### The national pension system in Denmark and early exit options

The Danish pension system is designed so that it is possible to receive a pension from multiple sources [31]. Normally, you will qualify for a state pension when you reach the state pension age, which in 2018 was 65 (i.e. at the study baseline) and 66 in 2020 (i.e. at study follow-up). For wage earners, the employer will also generally ensure that some of the salary is contributed to a pension savings account, and it is also possible to set up an individual pension scheme. Therefore, the Danish pension system is typically divided into three different kinds of pensions: the statutory pensions, the labour market pensions and the individual pensions. The statutory pensions are those pensions that the public sector controls, such as state pensions and disability pensions. There are also other pension schemes, such as ATP Livslang Pension (Lifelong Pension) which covers almost everyone. The labour market pensions are the pensions that are set up as part of the employment contract. The individual pensions are the schemes that are individually set up via a pension company or bank.

Disability pension is an early exit system for people with significant and permanent loss of work ability. The pension is permanent and compensates until state pension age. To qualify, an attempt to increase work ability must have been made without success. In Denmark it is not possible to receive the state pension before state pension age, regardless of state of health. But for individuals who are not able to work due to ill health it may be possible to receive senior pension. Senior pension gives people with many years of employment the opportunity to leave the labor market early. Additionally, there is a voluntary early retirement program (VERP) for people who have paid retirement contributions for at least 30 years and are members of an unemployment insurance fund. Early retirement pension means that individuals are entitled to retire 1, 2 or 3 years earlier if they fulfil the conditions for how many years they have been attached to the labour market (seniority).

### Control variables

The statistical model was controlled for the following potential confounding factors: age (years), gender (male/female), education (described below), body mass index (BMI: kg/m^2^), smoking status (No/Yes), and physical activity during leisure (described below).

Education was defined as the highest attained educational level and drawn from a national register handled by Statistics Denmark: 1) Primary school or high school (unskilled worker), 2) Vocational education (skilled worker), 3) Further education [32].

Physical activity during leisure was assessed by the following question: ‘How would you describe your physical activity level during leisure for the last 12 months?’. Respondents were given the following response options: 1) ‘Mostly sedentary’, 2) ‘Light exercise at least 4 h a week’, 3) ‘Sports or heavy physical activity at least 4 h per week’ and 4) ‘Training and competing regularly and several times a week’ [33, 34].

### Statistical analysis

Using the GLIMMIX procedure (PROC GLIMMIX, SAS version 9.4) with model-assisted weights, representative risk ratios (RR) for loss of paid employment were modelled as a function of each predictor variable within the 2-year follow-up period. Model-assisted weights were applied to ensure representativeness of the results by reducing the risk of bias due to differences in response rate and size between subgroups of the study population. The weights were applied based on the following background variables: gender, age group, occupational industry, highest completed education, family income, family type, and origin. The model-assisted weights were corrected using Statistics Denmark’s register’s information. The statistical model was adjusted for age, gender, highest completed education, and lifestyle (BMI, smoking status, physical activity during leisure). The estimates are presented as risk ratios (RR) with 95% confidence intervals (CI).

To evaluate potential age differences, an additional analysis was performed by stratifying the participants into two distinct age groups: 50–55 years and 56–63 years. Since poor health is a strong predictor of early labour market exit, an additional descriptive analysis was performed to illustrate potential differences and similarities in expected reasons for leaving the labour market between seniors with good and poor health. The respondents were stratified by self-rated health into two groups based on the question: ‘How do you rate your overall current health?’ and with the following response options: 1) ‘Excellent’, 2) ‘Very good’, 3) ‘Good’, 4) ‘Fair’, or 5) ‘Poor’?’. Response options were further dichotomized into ‘Good health’ (response option 1–3) and ‘Poor health’ (response option 4–5). The dichotomized self-rated health variable was crossed with the 15 expected reasons for leaving the labour market.

## Results

Table 1 illustrates the baseline characteristics of the study sample. Of the 10,320 workers, the mean age was 55.8 years and the proportion of women was 48%. Of the study sample, 1755 workers (weighted percentage = 12%) lost paid employment during the follow-up period from 2018 to 2020.

**Table 1:** Baseline characteristics of the study sample

	**Study sample**			
	N	Percent	Mean	SE
**Age, years**	10,320		55.8	0.043
**Gender**				
Women	4812	48		
Men	5508	52		
**Education**				
Unskilled worker	2043	21		
Skilled worker	4389	41		
Further education	3888	38		
**Smoking**				
No	8325	81		
Yes	1936	19		
**BMI**				
< 18	57	1		
18- < 25	4256	42		
25- < 30	4064	40		
30- < 35	1388	13		
35- < 40	321	3		
≥ 40	103	1		
**Physical activity during leisure**				
Seated	1494	14		
Light exercise at least 4 h per week	6221	61		
Sports or heavy physical activity at least 4 h per week	2358	23		
Training and competing regularly and several times a week	203	2		

Ordered from high to low, the most prevalent expected reasons for leaving the labour marker were (weighted percentage of workers): ‘That you want to determine yourself what you want to do’ (48%), ‘To have more time for hobbies’ (44%), ‘Possibility of receiving pension’ (29%), ‘That you will not be capable of doing your job’ (26%), ‘Possibility of receiving voluntary early retirement pension’ (22%), ‘Economic considerations’ (20%) and ‘Poor physical health’ (19%).

When stratifying by health status (i.e. good health; poor health), differences were observed in the proportion of participants mentioning the following factors as expected reasons for leaving the labour market: ‘That you want to determine yourself what you want to do’ (50%; 31%); ‘To have more time for hobbies’ (46%; 29%); ‘That you will not be capable of doing your job’ (24%; 42%); ‘Poor physical health’ (15%; 49%); ‘Poor mental health’ (5%; 10%); ‘Economic considerations’ (21%; 16%). For the remaining expected reasons, no or only minor differences were observed between seniors with good and poor health (< 3 percentage points).

Table 2 shows the prospective risk of expected reasons for leaving the labour market and loss of paid employment before state pension age. Expected reasons related to the domain of ‘Health, work demands and occupational well-being’ increased the risk of loss of paid employment: ‘Poor physical health’ (RR 1.47, 95% CI 1.45–1.49), ‘Poor mental health’ (RR 1.36, 95% CI 1.32–1.40), ‘That you will not be capable of doing your job’ (RR 1.20, 95% CI 1.18–1.22), and ‘That you do not thrive at the workplace’ (RR 1.14, 95% CI 1.11–1.17). ‘Possibility of receiving voluntary early retirement pension’ was also an expected reason that increased the risk of loss of paid employment (RR 1.30, 95% CI 1.28–1.32).

**Table 2:** Association between expected reasons for leaving the labour market and loss of paid employment before state pension age. RR = risk ratio CI = confidence interval

		**n**	**weighted %**	**RR (95% CI)**
**Leisure**				
That you want to determine yourself what you want to do	No	5321	51.6	1
	Yes	4997	48.4	0.87 (0.86–0.88)
To have more time for hobbies	No	5793	56.0	1
	Yes	4525	44.0	0.88 (0.87–0.89)
**Health, work demands and occupational well-being**				
That you will not be capable of doing your job	No	7671	73.9	1
	Yes	2647	26.1	1.20 (1.18–1.22)
Poor physical health	No	8317	81.0	1
	Yes	2001	19.0	1.47 (1.45–1.49)
That you do not thrive at the workplace	No	9407	91.2	1
	Yes	911	8.8	1.14 (1.11–1.17)
Poor mental health	No	9751	94.6	1
	Yes	567	5.4	1.36 (1.32–1.40)
**Economy and retirement considerations**				
Possibility of receiving pension	No	7416	71.1	1
	Yes	2902	28.9	0.77 (0.75–0.78)
Economic considerations	No	8245	79.6	1
	Yes	2073	20.4	0.72 (0.71–0.74)
Possibility of receiving voluntary early retirement pension	No	8013	77.6	1
	Yes	2305	22.4	1.30 (1.28–1.32)
Good retirement conditions at the workplace	No	9651	93.6	1
	Yes	667	6.4	1.02 (0.99–1.06)
**Norms**				
It is common to leave at that age in your type of work	No	9182	88.6	1
	Yes	1136	11.4	0.94 (0.92–0.97)
To make space for younger employees	No	9203	89.2	1
	Yes	1115	10.8	0.90 (0.88–0.92)
**External factors**				
Wish from spouse	No	9629	93.5	1
	Yes	689	6.5	0.90 (0.88–0.93)
Termination of employment	No	9807	95.5	1
	Yes	511	4.5	0.91 (0.88–0.95)
At the request of the workplace	No	10,077	97.7	1
	Yes	241	2.3	0.96 (0.92–1.01)
**None of the above**	No	10,026	97.2	1
	Yes	292	2.8	1.07 (1.03–1.12)

Adjusted for age, gender, education, lifestyle (BMI, smoking status, physical activity during leisure)

Expected reasons related to the domains of ‘Leisure’ and ‘Economy and retirement considerations’ decreased the risk of loss of paid employment: ‘That you want to determine youself what you want to do’ (RR 0.87, 95% CI 0.86–0.88); ‘To have more time for hobbies’ (RR 0.88, 95% CI 0.87–0.89), ‘Economic considerations’ (RR 0.72, 95% CI 0.71–0.74); and ‘Possibility of receiving pension’ (RR 0.77, 95% CI 0.75–0.78). Expected reasons related to the domain of ‘Norms’ (‘It is common to leave at that age in your type of work’; ‘To make space for younger employees’), and ‘External factors’ (‘Termination of employment relation’; ‘Wish from spouse’) were also associated with decreased risk of loss of paid employment before state pension age.

Table 3 shows the prospective risk of expected reasons for leaving the labour market and loss of paid employment before the state pension age stratified by two age groups: 50–55 years and 56–63 years. Several expected reasons related to the domain of ‘Health, work demands and occupational well-being’ predicted the risk of loss of paid employment to a greater extent among workers aged 50–55 compared to those aged 56–63. Specifically ‘Poor physical health’, ‘Poor mental health’ and ‘That you will not be capable of doing your job’.

**Table 3:** Age-stratified analysis on the association between expected reasons for leaving the labour market and loss of paid employment before state pension age. RR = risk ratio CI = confidence interval

		**50–55 yrs**			**56–63 yrs**		
		**n**	**weighted %**	**RR (95% CI)**	**n**	**weighted %**	**RR (95% CI)**
**Leisure**							
That you want to determine yourself what you want to do	No	2413	50.9	1	2908	52.4	1
	Yes	2336	49.1	0.79 (0.77–0.81)	2661	47.6	0.92 (0.91–0.94)
To have more time for hobbies	No	2611	55.0	1	3182	56.9	1
	Yes	2138	45.0	0.81 (0.79–0.83)	2387	43.1	0.92 (0.90–0.93)
**Health, work demands and occupational well-being**							
That you will not be capable of doing your job	No	3343	70.2	1	4328	77.5	1
	Yes	1406	29.8	1.53 (1.49–1.57)	1241	22.5	1.05 (1.03–1.07)
Poor physical health	No	3781	80.0	1	4536	82.1	1
	Yes	968	20.0	1.68 (1.63–1.72)	1033	17.9	1.35 (1.33–1.38)
That you do not thrive at the workplace	No	4378	92.3	1	5029	90.0	1
	Yes	371	7.7	0.98 (1.94–1.03)	540	10.0	1.22 (1.19–1.25)
Poor mental health	No	4467	94.3	1	5284	94.9	1
	Yes	282	5.7	1.65 (1.58–1.72)	285	5.1	1.21 (1.17–1.26)
**Economy and retirement considerations**							
Possibility of receiving pension	No	3224	67.4	1	4192	74.9	1
	Yes	1525	32.6	0.83 (0.80–0.85)	1377	25.1	0.73 (0.71–0.74)
Economic considerations	No	3739	79.0	1	4506	80.3	1
	Yes	1010	21.0	0.66 (0.63–0.68)	1063	19.7	0.76 (0.74–0.78)
Possibility of receiving voluntary early retirement pension	No	3968	83.2	1	4045	72.0	1
	Yes	781	16.8	0.99 (0.95–1.02)	1524	28.0	1.49 (1.46–1.52)
Good retirement conditions at the workplace	No	4395	92.7	1	5256	94.5	1
	Yes	354	7.3	0.84 (0.79–0.88)	313	5.5	1.17 (1.12–1.21)
**Norms**							
It is common to leave at that age in your type of work	No	4139	86.8	1	5043	90.4	1
	Yes	610	13.2	0.81 (0.78–0.84)	526	9.6	1.05 (1.02–1.08)
To make space for younger employees	No	4202	88.6	1	5001	89.7	1
	Yes	547	11.4	1.04 (1.00–1.08)	568	10.3	0.86 (0.84–0.89)
**External factors**							
Wish from spouse	No	4438	93.7	1	5191	93.3	1
	Yes	311	6.3	1.01 (0.96–1.07)	378	6.7	0.84 (0.81–0.87)
Termination of employment	No	4506	95.3	1	5301	95.7	1
	Yes	243	4.7	0.73 (0.68–0.78)	268	4.3	1.02 (0.97–1.06)
At the request of the workplace	No	4620	97.7	1	5457	97.8	1
	Yes	129	2.3	0.52 (0.46–0.58)	112	2.2	1.18 (1.12–1.25)
**None of the above**	No	4626	97.3	1	5400	97.0	1
	Yes	123	2.7	1.60 (1.51–1.70)	169	3.0	0.84 (0.80–0.89)

Adjusted for age, gender, education, lifestyle (BMI, smoking status, physical activity during leisure)

In contrast, reasons related to retirement consideration (i.e. ‘possibility of receiving voluntary retirement pension’ and ‘good retirement conditions at the workplace’) increased the risk for loss of paid employment only among older workers aged 56–63.

## Discussion

Expected reasons for leaving the labour market predicted actual labour market participation among older workers. Expected reasons related to ‘Health, work demands and occupational well-being’ and the possibility of receiving voluntary early retirement pension increased the risk of loss of paid employment before state pension age, whereas expected reasons related to ‘Leisure’, ‘Economy’ and ‘Norms’ decreased the risk. Age-stratified analyses indicated that expected reasons related to poor physical and mental health, and not being capable of doing the job are stronger PUSH-factors among workers aged 50–55 compared to those aged 56–63 (i.e. predicted risk of loss of paid employment to a greater extent).

For the entire study sample, factors related to the domains of ‘Leisure’, ‘Health, work demands and occupational well-being’ and ‘Economy and retirement decisions’ were the most salient expected reasons for leaving the labour market. Therefore, the discussion will mainly focus on the prospective association between these factors and loss of paid employment.

### Reasons associated with increased risk of loss of paid employment

Expected reasons related to the domain of ‘Health, work demands and occupational wellbeing’ significantly increased the risk of loss of paid employment before state pension age. The present analyses, therefore, validate that these are actual push factors. This domain includes both ‘Poor physical health’, ‘Poor mental health’, ‘That you do not thrive at the workplace’, and ‘That you will not be capable of doing your job’. These results are in line with previous studies showing associations between poor health (physical and mental) and premature exit from the labour market [1]. ‘Not being capable of doing your job’ reflects the balance between individual resources (including health) and work demands (both physical and psychosocial), i.e. work ability. Previous studies have identified high physical work demands as a push factor since it associates with the risk of sickness absence [10–13], early retirement [9, 14, 15], and disability pension [15–17]**,** especially among older workers [18]. Further, adverse psychosocial working conditions can also act as push factors, evidenced by studies in which low job control [16, 19–21] and low skill discretion [14] predicted early labour market exit. By contrast, positive psychosocial work factors such as high levels of influence and recognition from management act as stay factors that increase the likelihood of choosing to work beyond state pension age [22]. Thus, the balance between push and stay factors is important for the ability and willingness to work to a high age.

Not thriving at the workplace increased the risk of loss of paid employment. This factor may be related to poor psychological well-being [22, 35]. Von Bonsdorff et al. 2010 [35] found that factors related to the extent to which ageing employees of the Finnish metal industry and retail trade thrive at their workplace, i.e. poor work ability, frequent emotional exhaustion, low organizational commitment, and low job control were associated with increased prevalence of early retirement intentions. The authors concluded that by enhancing well-being, employees may choose to stay at work for longer rather than retire early.

Identifying the possibility of receiving a voluntary early retirement pension (VERP) as an expected reason for retirement also increased the risk of losing paid employment. VERP has been considered a factor that pulls people out of the labour market (i.e. pull factor). However, it may also represent a possible exit route from the labour market for those experiencing strong push factors, such as exposure to physically demanding work and/or adverse psychosocial working conditions. In line with this, it has previously been suggested that workers (50–60 years) with deteriorating health may choose to wait for VERP benefits instead of applying for disability pension [25, 26]. A study from the Netherlands showed that a pension form to remove the possibility to receive early retirement benefits, led to increased work participation for the age group in question, but at the same time led to higher unemployment levels [36]. The current results also indicate that mentioning VERP as an expected reason for leaving employment can be used to predict future labour market participation among older employees. This is in line with a previous study on older Danish workers showing that access to early retirement benefits through the VERP predicted early retirement among workers with both physically demanding and sedentary work [24].

### Reasons associated with reduced risk of loss of paid employment

Expected reasons related to the domains of ‘Leisure’, e.g. ‘That you want to determine yourself what you want to do’; ‘To have more time for hobbies’, and ‘Economy’, e.g. ‘Economic considerations’; ‘Possibility of receiving pension’, significantly decreased the risk of losing paid employment before state pension age.

For the present study, a smaller proportion of seniors with good self-rated health mentioned factors related to ‘Health and work demands’ (i.e. push factors) as expected reasons for leaving the labour market, compared to seniors with poor health. Thus, it seems that senior workers with good health to a lesser extent expect health problems as a reason for leaving the labour market and, therefore, report other expected reasons for leaving. Such expected reasons could be related to economy and leisure, which may explain why these factors are associated with a reduced risk of loss of paid employment before the state pension age. When interpreting the results, it is also important to bear in mind that the outcome variable represents loss of paid employment before the official state pension age. The factors investigated therefore relates to premature loss of paid employment, while other expected reasons could be important for leaving employment at or after the official state pension age.

Expected reasons related to more time for hobbies and self-determination reduced the risk of loss of paid employment. Existing studies on retirement patterns of older workers have shown that intentions to retire are conditioned by a preference for more leisure time to be used for a new ‘life project’ and/or social gains such as spending more time with grandchildren [27]. In line with this, the wish for more time for leisure activities has previously been recognized as a jump factor and has been described as a motivation for early retirement [28]. Further, flexible working hours have also been identified as an incentive for prolonging working life [37], since it may promote a good work-life balance.

Expected reasons related to economy also reduced the risk of loss of paid employment. Research has pointed to the relationship between personal economy and the intention to both retire or continue working. A previous study on senior workers identified financial incentives, i.e. if it would pay off better economically, as one of the most prevalent expected reasons to prolong working life [23]. However, participants expecting to retire before state pension age were less likely to mention this factor compared to those expecting to retire at or after state pension age. Thus, it seems that other factors could be more important for this group of workers. In line with this, it has been speculated that personal economy may act as both a pull, stuck and stay factor, i.e. the worker may be (i) pulled out due to an attractive retirement scheme, (ii) stuck at the labour market because the personal economy does not allow one to retire despite poor health, (iii) tempted to stay longer and thereby gain further income and pension savings [3].

### Age-specific differences

The results stratified by age-group revealed that several expected reasons related to the domain of ‘Health, work demands and occupational well-being’ predicted the risk of loss of paid employment to a greater extent among workers aged 50–55. Thus, it seems that PUSH factors (e.g. poor physical and mental health, and not being capable of doing your job) are relatively stronger among the younger compared to the older age group of senior workers. This seems reasonable, since the oldest age group – all else being equal – will be more likely to leave the labour market since they are closer to the official state pension age. Further, a more pronounced ‘healthy worker effect’ among the oldest senior workers could also have influenced this finding.

The ‘possibility for receiving voluntary retirement pension’ (VERP) and ‘good retirement conditions at the workplace’ increased the risk for loss of paid employment only among older workers aged 56–63. VERP has previously been considered a factor that pulls people out of the labour market, but it may also be a way out of the labour market for those experiencing strong push factors, for example, exposure to physically demanding work and/or adverse psychosocial working conditions. In line with this, it has previously been suggested that senior workers (50–60 years) with deteriorating health may choose to wait for VERP benefits instead of applying for disability pension [24–26]. This could potentially explain why the possibility of receiving VERP was not associated with an increased risk of losing paid employment among the younger group of senior workers.

### Practical implications

Overall, it seems that asking older workers, years before state pension age, about expected reasons for leaving the labour market can provide knowledge warranted for designing preventive strategies aiming at increasing or maintaining future labour market participation. Specifically, seniors identifying factors related to ‘Health, work demands and occupational well-being’ as expected reasons for leaving, seem to be at increased risk of losing paid employment before state pension age and should therefore be the target for preventive workplace efforts. Thus, preventing early labour market detachment should take the worker’s expected reasons for leaving into account. Expected reasons for leaving could for instance be discussed and identified through systematic dialogues at the workplace between the worker and employer years before the official state pension age Based on the stratified analyses, it seems especially important to focus on preventive strategies among the young group of older workers. The differences between a 50-year-old and a 60-year-old with regard to reasons for retirement – and its association with actual labour market participation – seem to be quite diverse. For instance, mentioning poor physical and mental health (along with not being capable of doing the job) as expected reasons for retirement have a stronger push-effect among the younger compared to the older group of senior workers. Thus, it seems pivotal for workplaces and decision-makers to focus on early identification of expected reasons for retirement to better target and develop preventive efforts to keep a larger proportion of older workers in the labour market.

### Strengths and limitations

The study has both strengths and limitations. Using a weight variable based on national registers is a strength, as this makes the estimates representative of older workers in Denmark. Further, drawing the random sample among all eligible people in Denmark, ensures that 1) all wage earners above 50 years in Denmark can be selected, 2) that this selection is made by drawing lots, and 3) that the probability of being selected by these lots is known. The above procedures ensured that the employed data is representative of older workers in Denmark (> 50 years), which strengthens the generalizability of the present results [3, 29].

Another strength is that the outcome measure was derived from a national register containing high-quality daily information on wage and social transfer payments for all Danish residents. This procedure eliminates any potential self-reporting bias in the assessment of loss of paid employment.

It is a limitation that our data does now allow categorizing our outcome measure into different routes of loss of paid employment such as disability pension, unemployment, early voluntary retirement or reduced working hours. The predictive value of expected reasons for leaving the labour market could differ between routes of loss of paid employment. Importantly, the outcome measure is loss of paid employment before state pension age, and not actual retirement. For this reason, we set an upper limit of participant age (63 years) to avoid participants reaching state pension age during follow-up. However, leaving the labour market or substantially reducing the working hours before the state pension age is likely to reflect involuntary push mechanisms, but some may also have lost paid work due to voluntary early retirement. However, to test this, a future prospective follow-up study is needed to determine the influence of retirement intentions on different routes of retirement.

The applicability of the results to other countries with different arrangements of the pension system and labor market protection needs to be further investigated. The generalizability of the present study, therefore, seems to apply to workers in Denmark (i.e. a welfare state) within the age range of 50–63 years.

Another limitation to the study is that the observational study design does not allow causal inferences. Further, it was not possible to identify if a proportion of participants left paid employment gradually though self-employment as a first step towards retirement since the registers do not hold information on how many hours self-employed work per week.

## Conclusions

Expected reasons for leaving the labour market were prospectively associated with actual labour market participation among older workers. Expected reasons related to ‘Health, work demands and occupational well-being’ and the possibility of receiving voluntary early retirement pension increased the risk of loss of paid employment before state pension age, whereas expected reasons related to ‘Leisure’, ‘Economy’ and ‘Norms’ decreased the risk. Importantly, expected reasons related to poor physical and mental health, and not being capable of doing the job seem to be stronger PUSH-factors among workers aged 50–55 compared to those aged 56–63. Preventing early labour market detachment should take the worker’s expected reasons for leaving into account.

## Data Availability

The authors encourage collaboration and use of the data by other researchers. Data is stored on the server of Statistics Denmark, and researchers interested in using the data for scientific purposes should contact the project leader Prof. Lars L. Andersen, LLA@NFA.dk.
